# Synthesis, Pharmacological Evaluation, and Computational Studies of Cyclic Opioid Peptidomimetics Containing β^3^-Lysine

**DOI:** 10.3390/molecules27010151

**Published:** 2021-12-28

**Authors:** Karol Wtorek, Piotr F. J. Lipiński, Anna Adamska-Bartłomiejczyk, Justyna Piekielna-Ciesielska, Jarosław Sukiennik, Alicja Kluczyk, Anna Janecka

**Affiliations:** 1 Department of Biomolecular Chemistry, Medical University of Lodz, Mazowiecka 6/8, 92-215 Lodz, Poland; karol.wtorek@umed.lodz.pl (K.W.); anna.adamska@umed.lodz.pl (A.A.-B.); justyna.piekielna@umed.lodz.pl (J.P.-C.); 2 Department of Neuropeptides, Mossakowski Medical Research Institute, Polish Academy of Sciences, Pawińskiego 5, 02-106 Warsaw, Poland; plipinski@imdik.pan.pl; 3 TriMen Chemicals Ltd., 92-318 Lodz, Poland; sukiennik@trimen.pl; 4 Faculty of Chemistry, University of Wroclaw, 50-383 Wroclaw, Poland; alicja.kluczyk@chem.uni.wroc.pl

**Keywords:** opioid receptors, β-amino acids, peptide synthesis, receptor binding studies, functional assay

## Abstract

Our formerly described pentapeptide opioid analog Tyr-c[D-Lys-Phe-Phe-Asp]NH_2_ (designated **RP-170**), showing high affinity for the mu (MOR) and kappa (KOR) opioid receptors, was much more stable than endomorphine-2 (EM-2) in the rat brain homogenate and displayed remarkable antinociceptive activity after central (intracerebroventricular) and peripheral (intravenous ) administration. In this report, we describe the further modification of this analog, which includes the incorporation of a β^3^-amino acid, (*R*)- and (*S*)-β^3^-Lys, instead of D-Lys in position 2. The influence of such replacement on the biological properties of the obtained analogs, Tyr-c[(*R*)-β^3^-Lys-Phe-Phe-Asp]NH_2_ (**RP-171**) and Tyr-c[(*S*)-β^3^-Lys-Phe-Phe-Asp]NH_2_, (**RP-172**), was investigated in vitro. Receptor radiolabeled displacement and functional calcium mobilization assays were performed to measure binding affinity and receptor activation of the new analogs. The obtained data revealed that only one of the diastereoisomeric peptides, **RP-171**, was able to selectively bind and activate MOR. Molecular modeling (docking and molecular dynamics (MD) simulations) suggests that both compounds should be accommodated in the MOR binding site. However, in the case of the inactive isomer **RP-172**, fewer hydrogen bonds, as well as instability of the canonical ionic interaction to Asp^147^, could explain its very low MOR affinity.

## 1. Introduction

Among the three opioid receptors, mu (MOR), delta (DOR), and kappa (KOR), MOR plays the most important role in the modulation of pain signals and, therefore, is an important target in medicinal chemistry and drug development [1]. The two endogenous compounds activating MOR are endomorphin-1 (EM-1, Tyr-Pro-Trp-Phe-NH_2_) and endomorphin-2 (EM-2, Tyr-Pro-Phe-Phe-NH_2_) [2]. Over the years, numerous chemical modifications of these ligands have been reported in order to provide specific information on their structure–activity relationship and to find drug candidates with improved therapeutic properties [3,4,5]. Among various modifications of opioid peptides, cyclization of their linear structures was used to restrict flexibility and to obtain better-defined conformations, allowing for the identification of receptor binding sites [6,7,8,9].

Endomorphins are very short peptides lacking reactive side chain groups, which makes their cyclization difficult. One of the structural elements considered essential for their binding to MOR is the free cationic amino group of Tyr^1^ [10,11,12], and this feature does not encourage head-to-tail cyclization. In order to obtain cyclic analogs based on the structure of EM-2 but still to preserve the free N-terminal amino group, we introduced into the sequence of EM-2 additional amino acids with functionalized side chains. A pentapeptide analog Tyr-c[D-Lys-Phe-Phe-Asp]NH_2_ (designated **RP-170**), in which cyclization was achieved through the amide bond between D-Lys and Asp side chains, displayed high affinity for MOR, was much more stable than EM-2 in the rat brain homogenate and showed remarkable antinociceptive activity after central (i.c.v.) and peripheral (i.v.) administration [13]. The presence of a D-amino acid in position 2 (as in opioid peptides isolated from amphibian skin) was shown to enforce a different conformation of a peptide, greatly improving MOR binding as compared with Tyr-c[Lys-Phe-Phe-Asp]NH_2_ [14]. Molecular docking studies of **RP-170** revealed that the amino group of Tyr^1^ provided ionic interactions with Asp^147^ residue in the transmembrane helice TM III of the receptor, while Asp amide effectively interacted with Asp^216^ and Cys^217^ belonging to the extracellular loop EL II. The presence of a Lys residue allowed for the formation of another strong interaction between Asp^147^ and Lys-NH [15].

Further modifications of **RP-170** produced analogs with different opioid receptor preferences. Introduction of Dmt instead of Tyr^1^ increased cyclopeptide affinity to MOR [16]. The reduction in the ring size increased MOR selectivity [17]. Substitution of the Phe residues by amino acids fluorinated in the aromatic ring (4-F-Phe, 2,4-diF-Phe, 4-CF_3_Phe) produced either high-affinity MOR/KOR agonists, non-selective MOR/DOR/KOR agonists, or selective KOR agonists [18], indicating that even small modifications in the side chains can completely change their orientation in the receptor cavity.

In the present study, we investigated the influence of a β-amino acid on the biological properties of **RP-170**. D-Lys was replaced by (*R*)- or (*S*)-β^3^-Lys, obtained by homologation of D- or L-ornitine (Orn). This modification produced compounds isomeric to **RP-170** with the same size of the macrocyclic ring (17-membered), as in the parent peptide. Opioid receptor binding and activation were studied, and the obtained results were rationalized by molecular docking and molecular dynamics (MD) simulations.

## 2. Results

### 2.1. Synthesis of Protected (R)- and (S)-β^3^-Lys

**Scheme 1:**
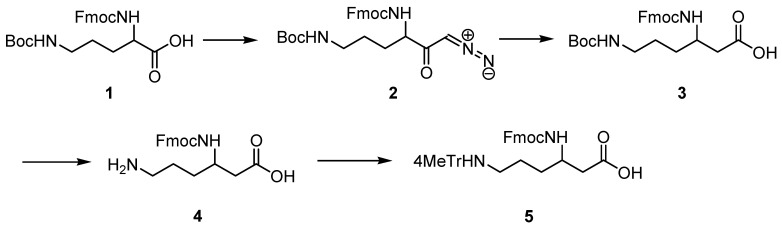
Synthesis of Fmoc-(R)- and (S)-β^3^-Lys(Mtt).

(R)- and (S)-Fmoc-β^3^-Lys (Mtt), which are not available commercially, were obtained by homologation of D- and L-Orn, respectively, according to the general procedure [19]. The synthetic protocol is outlined in Scheme 1.

### 2.2. Synthesis of Cyclopeptides

**Figure 1:**
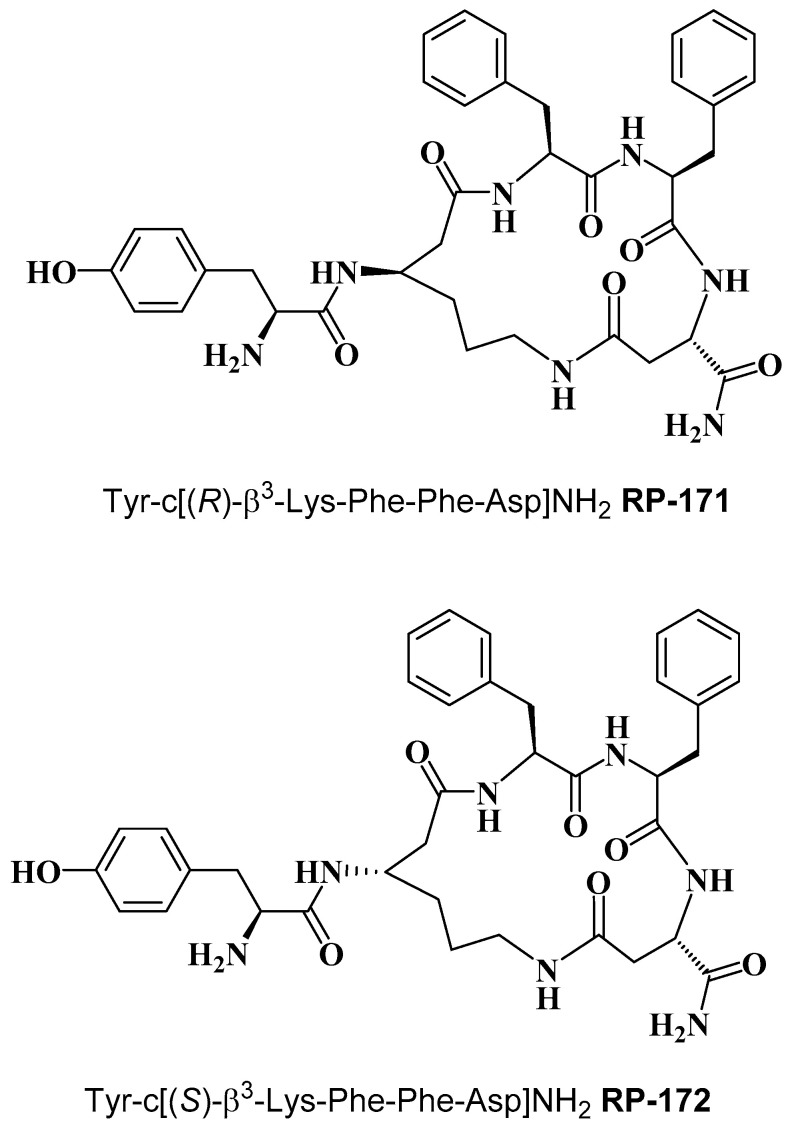
Chemical structure of **RP-171** and **RP-172**.

Cyclopeptides containing a β-amino acid, Tyr-c[(R)-β^3^-Lys-Phe-Phe-Asp]NH_2_ (**RP-171**) and Tyr-c[(S)-β^3^-Lys-Phe-Phe-Asp]NH_2_ (**RP-172**) (Figure 1) were synthesized on the solid support, using Fmoc/t-Bu strategy, with the hyper-acid labile groups (Mtt and O-2-PhiPr) for the selective protection of amine/carboxyl side chains of (R)- and (S)-β^3^-Lys and Asp, engaged in cyclization. After deprotection of the functionalized side chains, the linear sequences were cyclized through amide bond formation. Final products were obtained with a purity greater than 95%, as assessed by semi-preparative RP-HPLC. The detailed analytical data of the synthesized peptides are provided in the Supplementary Materials (Table S1, Figures S2 and S3).

### 2.3. LC-MS, LC-MS^n^, and Quantum Chemical Calculation Studies

**Figure 2:**
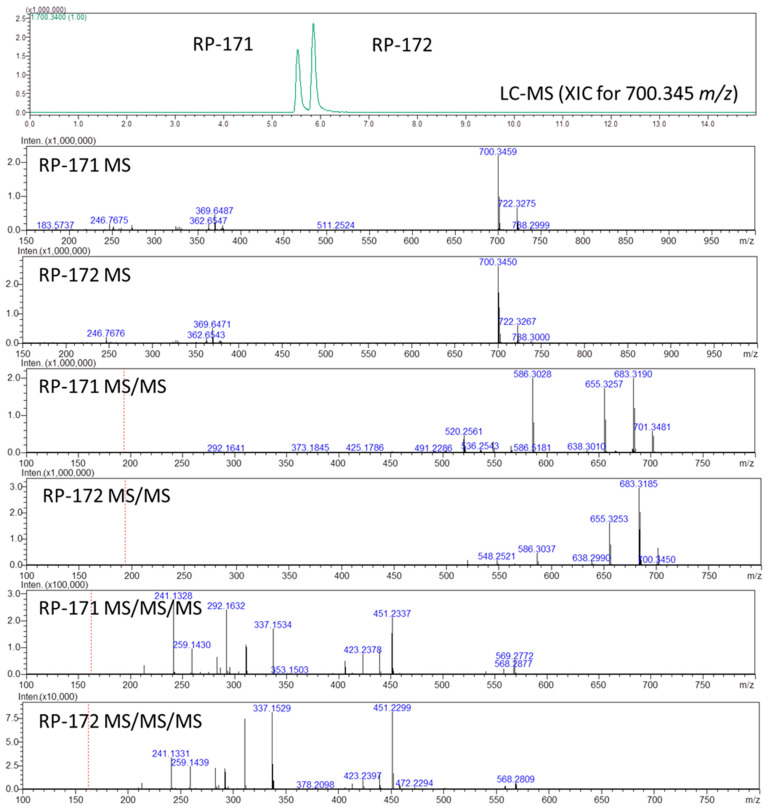
LC-MS^n^ analysis of peptides **RP-171** and **RP-172**. Extracted ion chromatogram (XIC) showing two peaks for the mixture of peptides (top panel): MS panels, all spectra recorded for the retention time of indicated peaks; MS spectra, MS/MS spectra for the precursor ions *m*/*z* 700.34, and MS/MS/MS spectra for the precursor ions *m*/*z* 586.30.

During the routine LC-MS analysis of analogs **RP-171** and **RP-172**, we noticed a distinct difference in retention times and MS^n^ patterns for these diastereoisomeric peptides. To confirm our observation, we subjected a mixture of these peptides to LC-MS and MS^n^ experiments. The HPLC analysis in reversed-phase mode revealed that the isomeric peptides separate easily, using both C_18_ column (Aeris Peptide) and biphenyl column (Kinetex Biphenyl), known for additional π-π interactions [20], with a nearly 0.5 min retention time difference in both cases in a 10 min gradient run from 5 to 80% acetonitrile in water (Figure 2 and Figures S4–S6). Such a difference in retention time suggests altered interactions with the stationary phase, probably due to the shape of the molecules. It is interesting that the elution order from the biphenyl column was the same as from the C_18_ column.

To assign the order of isomeric peptides in the LC-MS experiment on the **RP-171** and **RP-172** peptide mixture (Figure 2), we used retention times obtained during analysis of pure peptides, supported by MS^n^ spectra. As expected, the MS spectra of peptides **RP-171** and **RP-172** were identical (panels **RP-171** MS and **RP-172** MS), and the difference in their collision-induced dissociation (MS^2^, panels **RP-171** MS/MS, and **RP-172** MS/MS) was related to an intensity of 586 *m*/*z* fragment ion. To find a more reliable distinction, the MS^3^ spectra were obtained for the precursors 586 from MS^2^ spectra (panels **RP-171** MS/MS/MS and **RP-172** MS/MS/MS, MS^3^ discussion in the Supplementary Materials).

The fragment ions observed in the MS/MS spectra were typical for peptide amides (consecutive loss of ammonia, 683 *m*/*z*, and carbon monoxide 655 *m*/*z*), whereas the 586 ion resulted from ring-opening and removal of the Asp residue. The difference in intensity of the 586 *m*/*z* ions in panels **RP-171** MS/MS and **RP-172** MS/MS suggests that the fragmentation of peptide **RP-171** occurs easier than in the case of **RP-172**, suggesting that peptide **RP-172** containing (S)-β^3^-Lys is more stable. 

**Figure 3:**
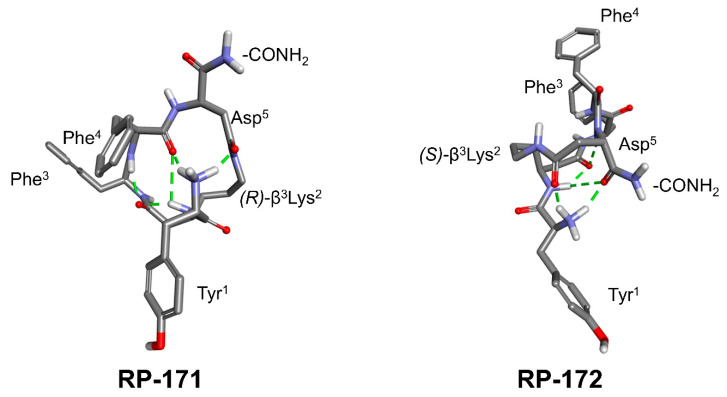
The lowest-lying conformers of **RP-171** and **RP-172** (at the B3LYP/6-31G(d,p) in the gas phase. Green dots show intramolecular hydrogen bonding.

This observation corresponds to the results of quantum chemical calculations (performed with Gaussian09 [21], Table S2) for both isomers. The lowest-lying (at the B3LYP/6-31G(d,p) level) gas-phase conformer of the [(S)-β^3^-Lys^2^]- analog is more stable by 3.5 kcal/mol (ΔG_298_) than the lowest-lying conformer of the [(R)-β^3^- Lys]- analog. The structures differ with respect to the intramolecular hydrogen bonds present (Figure 3). In the [(R)-β^3^-Lys]- analog, the Tyr^1^ amino group interacts with the backbone carbonyl oxygens of Phe^4^ and Asp^5^. This arrangement might facilitate internal cyclization upon the Asp residue loss. On the other hand, in **RP-172**, the Tyr^1^ amino group interacts with the carbonyl oxygens of (S)-β^3^-Lys^2^ and of the exocyclic CONH_2_.

### 2.4. Receptor Binding and Functional Activity

The binding affinities of cyclopeptides **RP-171** and **RP-172** toward MOR, DOR, and KOR were determined by competitive binding against [^3^H]DAMGO, [^3^H][D-Ala^2^]deltorphin-2, and U-69593, respectively, using membranes of CHO cells transfected with opioid receptors and are summarized in Table 1.

The parent compound **RP-170** displayed subnanomolar affinity to MOR, nanomolar to KOR, and did not show substantial DOR affinity. Replacement of D-Lys with (R)-β^3^-Lys generated **RP-171**, which showed about 50-fold lower affinity for MOR but did not bind to the other two opioid receptors, which made this analog much more selective. The diastereoisomeric **RP-172**, incorporating (S)-β^3^-Lys, did not bind to any of the three opioid receptors, showing that affinity of these analogs depended on the configuration of the β-amino acid.

The functional activities of the cyclopeptides in vitro were assessed at all three opioid receptors in calcium mobilization assay in which CHO cells co-expressing human recombinant opioid receptors and chimeric G proteins were used to monitor changes of intracellular calcium levels, reflecting activation of the G protein-coupled receptors (GPCR) [22,23].

The obtained results are summarized in Table 2. Agonist potencies of peptides are given as the negative logarithm of the molar concentration of an agonist that produces 50% of the maximal possible effect (pEC_50_). Ligand efficacy was expressed as intrinsic activity (α). Dermorphin, DPDPE, and dynorphin A were used as standard agonists for calculating efficacy at MOR, DOR, and KOR, respectively. In CHO-MOR cells, the parent analog **RP-170** induced a significant concentration-dependent release of Ca^2+^ ions (pEC_50_ = 8.93, α = 1.00), with efficacy and potency even higher than those of dermorphin (pEC_50_ = 8.57, α = 1.00). For peptides **RP-171** and **RP-172**, the calculated pEC_50_ values were 6.87 and 5.45, respectively (for concentration-response curves, see Figure S7). In CHO-DOR cells, DPDPE elicited a strong concentration-dependent Ca^2+^ release, showing high potency and maximal effect (pEC_50_ = 7.23, α = 1.00), while all three cyclopeptides were inactive. In CHO-KOR cells, dynorphin A induced a significant concentration-dependent Ca^2+^ release (pEC_50_ = 9.04, α = 1.00). The potency of **RP-170** was only slightly lower, showing high potency and maximal effect (pEC_50_ = 8.60, α = 1.00), **RP-171** displayed significantly lower potency but high efficacy (pEC_50_ = 5.99, α = 0.82), and **RP-172** was inactive. Summing up, in this assay, **RP-171** had similar receptor preferences as the parent **RP-170,** while **RP-172** was completely inactive, which points to the importance of the R-chirality at position 2 of these cyclopeptides.

### 2.5. Molecular Modeling

In order to obtain insight into the structural basis for the observed affinities, the analogs **RP-171** and **RP-172** were docked into the structure of the activated MOR (PDB accession code: 6DDF [24]) using AutoDock 4.2.6 [25]. The best scored poses were then subjected to molecular dynamics (MD) simulations (100 ns production, see Figure S8 for RMSD plots).

**Figure 4:**
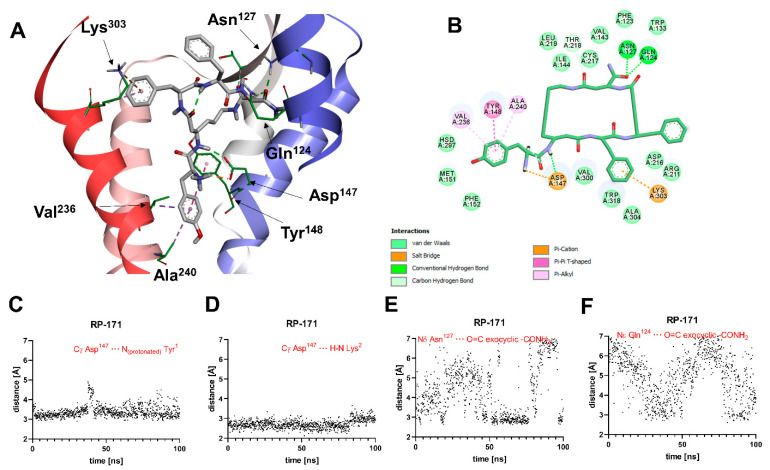
(**A**) Binding mode of **RP-171** (white sticks) in the MOR binding site, as found at t = 100.0 ns of the MD production run. The receptor is shown in a simplified manner, with only selected helices (ribbons) and side chains (thin sticks) shown. Display of nonpolar hydrogens is suppressed for clarity. (**B**) Diagram showing interactions between **RP-171** and the MOR binding site (interaction types colored according to the legend). (**C**–**F**) Time evolutions of selected distances associated with protein–ligand interactions during the MD simulations.

A general view of the binding pose of **RP-171**, as found in the MD simulations at t = 100.0 ns, is shown in Figure 4A. The interaction scheme is presented in Figure 4B. The compound is anchored in the MOR binding site first and foremost by the canonical interaction of the protonated amino group of Tyr^1^ with Asp^147^. Additionally, the amide hydrogen of the peptide bond joining Tyr^1^ and β^3^-Lys^2^ interacts with Asp^147^. These two interactions are stable throughout the simulation (Figure 4C,D). Other polar contacts stabilizing the complex are hydrogen bonds between the exocyclic carbonyl oxygen and Gln^124^ or Asn^127^, but these interactions fluctuate in the simulation time (Figure 4E,F). The remaining contacts are of apolar character. The aromatic ring of Tyr^1^ is involved with π-π stacking with Tyr^148^ and π-alkyl interactions with Ala^240^ and Val^236^ side chains. Other residues in the close vicinity of this aromatic ring are Met^151^ and His^297^. The aromatic ring of the Phe^3^ residue approaches Trp^318^, Lys^303^, and Ala^304^, while the Phe^4^ aromatic ring is exposed to the solvent close to the extracellular outlet of the binding site. For other residues participating in van der Waals contacts, refer to Figure 4B.

**Figure 5:**
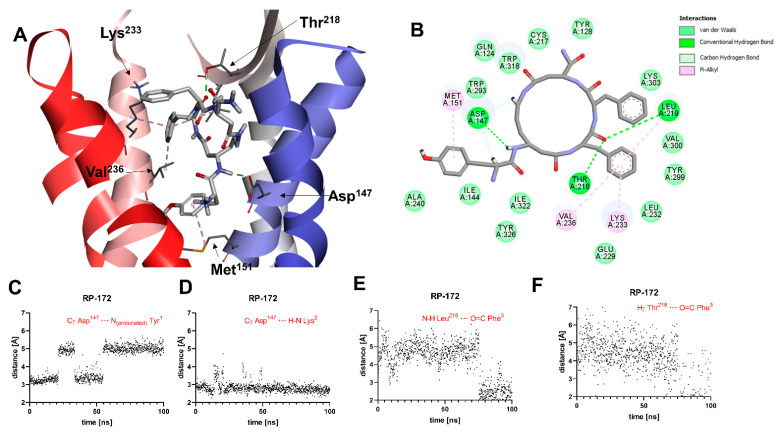
(**A**) Binding mode of **RP-172** (white sticks) in the MOR binding site, as found at t = 100.0 ns of the MD production run. The receptor is shown in a simplified manner, with only selected helices (ribbons) and side chains (thin sticks) shown. Display of nonpolar hydrogens is suppressed for clarity. (**B**) Diagram showing interactions between **RP-172** and the MOR binding site (interaction types colored according to the legend). (**C**–**F**) Time evolutions of selected distances associated with protein–ligand interactions during the MD simulations.

A general view of the binding pose of **RP-172**, as found in the MD simulations at t = 100.0 ns, is shown in Figure 5A. The interaction scheme is presented in Figure 5B. The only polar contact that is consistently present throughout the whole MD production is the H-bond interaction of amide hydrogen of the peptide bond joining Tyr^1^ and β^3^-Lys^2^ interacts with Asp^147^ (Figure 5D). Contrary to what was found for **RP-171**, and contrary to what would be expected for strong MOR agonists, the interaction between the protonated amino group of Tyr^1^ and Asp^147^ is unstable (Figure 5C). This H-bond, while present in the binding pose found by docking, is broken after the 65 ns of the MD simulations. Another polar contact that is broken during the MD run involves the interaction of exocyclic carbonyl oxygen with the Arg^211^ side chain guanidine group. By the end of the simulation, the carbonyl oxygen of Phe^3^ starts with backbone amide hydrogen of Leu^218^ and hydroxyl hydrogen of Thr^219^ (Figure 5E,F). With respect to apolar contacts (found in the final snapshots of the simulation), the Tyr^1^ aromatic ring interacts with the Met^151^ side chain (π-alkyl interaction). The Phe^3^ aromatic ring approaches Trp^318^ and participates in π-alkyl interactions with the side chains of Leu^219^, Lys^233^, and Val^236^. Other receptor residues interacting with the peptide are shown in Figure 5B.

## 3. Discussion

β-Amino acids, although much less abundant than their α-analogs, are also present in nature and exhibit interesting pharmacological properties. The difference between α- and β-amino acids is in the number of carbon atoms (one or two, respectively) that separate an amino and a carboxy termini. β-Amino acids with side chains other than H can exist as *R* or *S* isomers at either the α (C2) carbon or the β (C3) carbon, producing β^2^- or β^3^-amino acids, respectively.

The most common naturally occurring β-amino acid is β-alanine, which is a component of pantothenic acid (vitamin B_5_), which, in turn, is a component of coenzyme A. Another example of a natural β-amino acid is (1*R*,2*S*)-2-aminocyclopentanecarboxylic acid (cispentacin), an antifungal antibiotic isolated from *Bacillus cereus* [26].

β-Peptides (made of only β-amino acids) in general do not appear in nature, though among the opioid peptides, there are examples of such synthetic analogs [27]. More often mixed α/β-peptides, in which one or more β-residues are incorporated instead of some α-amino acids, were constructed [28,29,30,31,32,33,34].

An important advantage of peptide analogs incorporating β-amino acids over natural peptides is their stability against proteolytic degradation [35,36], which makes β-amino acids desirable building blocks in the preparation of peptide-based drugs [37].

In this report, we used (*R*)- and (*S*)-β^3^-Lys to assess the influence of a β-amino acid on the conformation of the macrocycle of Tyr-c[D-Lys-Phe-Phe-Asp]NH_2_ (**RP-170**), which has nanomolar MOR and KOR affinity. The obtained diastereoisomeric analogs **RP-171** and **RP-172** are also isomers of the parent compound **RP-170**, with which they share the same number of atoms in the whole structure and in the macrocycle. The difference between **RP-171**/**RP-172** and the parent **RP-170** is the point at which the exocyclic Tyr^1^ is attached to the ring (one carbon atom shift as compared with **RP-170**). The experimental evaluation of the binding affinity and functional activity of **RP-171** showed that such minor structural change had a significant effect on the biological properties causing 53- and 276-fold loss of affinity for MOR and KOR, respectively, as compared with the parent. The inversion of the configuration of β^3^-Lys in **RP-172** induced an almost complete loss of affinity of this peptide for the opioid receptors.

The diastereomers **RP-171** and **RP-172** exhibit slightly different lipophilicity, as could be seen from their chromatographic behavior in the reversed-phase liquid chromatography on a C_18_ column. No additional effects were observed when the biphenyl stationary phase was used, which may suggest that the arrangement of aromatic rings in the isomers was not suitable for interactions with a biphenyl motif.

Further differences between isomers were revealed after a thorough analysis of fragmentation patterns in the MS^n^ experiments. Peptide **RP-172** containing (*S*)-β^3^-Lys turned out to be more stable, and this observation corresponds with the results of conformational analysis and quantum chemical calculations. They show a significant difference in the structure and the energetics of the lowest-lying conformers of both diastereoisomers.

In our former work [33], we devised an interaction model for **RP-170** and its analogs, in which the peptides were anchored in the MOR binding pocket by interactions at the three key binding subsites. According to that model, in the S1 subsite, the protonable amino group of Tyr^1^ interacts with Asp^147^ (a typical contact for high-affinity MOR agonists of both peptide [24] and non-peptide character [38]). In the S2 and S3 subsites reside the aromatic rings of the Phe residues. Our analyses suggested that the ability to place Tyr^1^, Phe^3^, and Phe^4^ in these subsites is important for high MOR affinity.

The present results seem to corroborate this model. Replacement of D-Lys by (R)- or (S)-β3-Lys produced a topographical shift of Phe^3^ and Phe^4^ in regard to Tyr^1^. As a conse-quence (according to the molecular docking and molecular dynamics), neither **RP-171** nor **RP-172** could accommodate their Phe^3^ and Phe^4^ aromatic rings in the way the parent compound did. This explains the lower MOR affinity of **RP-171**. This compound exhibits, however, the canonical interaction between the Tyr^1^ amino group and Asp^147^. On the contrary, for **RP-172**, such interaction (while present in the docked pose) is unstable in the MD simulations. This could be correlated to a much-diminished MOR affinity found experimentally for this analog. The obtained experimental and theoretical data form the basis for further work on **RP-170** analogs, an important element of which will be ADME/T evaluation.

## 4. Materials and Methods

### 4.1. Materials

All protected α-amino acids were purchased from Bachem A (Bubendorf, Switzerland). Opioid radioligands, [^3^H]DAMGO, [^3^H]deltorphin-2, and [^3^H]U-69593, and human recombinant opioid receptors were purchased from PerkinElmer (Krakow, Poland). GF/B glass fiber strips were obtained from Whatman (Brentford, UK). Purity of peptides was determined by RP-HPLC and exact mass. Analytical and semi-preparative RP-HPLC was performed using Waters Breeze instrument (Milford, MA, USA) with dual absorbance detector (Waters 2487, MA, USA). All ESI-MS experiments were performed on a Shimadzu IT-TOF mass spectrometer (Shimadzu, Japan) equipped with ESI source connected to Nexera HPLC system (Shimadzu, Japan). The instrument was operated in the positive-ion mode. Peptide solutions (1 μL) were introduced in a 0.2 mL/min flow of mobile phase. For LC-MS experiments, Aeris Peptide C_18_ and Kinetex Biphenyl (Phenomenex, Torrance, CA, USA) were used, in a gradient reversed-phase mode, from 5 to 80% acetonitrile in water (both containing 0.1% HCOOH). ^1^H NMR spectra were recorded on a 500 MHz Brucker instrument in DMSO-d_6_, using residual DMSO as a resonance reference at 2.5 ppm.

### 4.2. Synthesis of Fmoc-Protected (R)- and (S)-β^3^-Lys(Mtt)

To the 500 mL three-necked, round bottom flask with Liebig’s condenser equipped with thermometer, magnetic stirrer and protected from moisture with a tube with anhydrous calcium chloride, a solution of Fmoc-D-Orn-(Boc)-OH (**1**) (3 g, 6.6 mmol, 1 eq) in 50 mL of tetrahydrofuran (THF) was added, stirred and cooled to −30 °C. Then, N-methylmorpholine (1.52 mL, 13.9 mmol, 2.1 eq) was added, followed by methyl chloroformate (0.56 mL, 7.3 mmol, 1.1 eq) added dropwise, and stirring was continued for 30 min at −30 °C. Next, the diazomethane obtained, using standard procedure, from Diazald® (8.48 g, 13.9 mmol, 6 eq.) was distilled along with diethyl ether directly to the flask. The temperature in the flask was maintained below −10 °C, and after 1 h, the cooling bath was removed. The reaction was completed in 2 h (LC-MS analysis). Acetic acid (5 mL) was added to decompose the excess diazomethane, and stirring was continued for 30 min. Then, 100 mL of diethyl ether was added, and the solution was washed with water (2 × 100 mL), 5% NaHCO_3_ (2 × 50 mL), and brine. The organic fraction was dried over MgSO_4_ to obtain, after evaporation, 3 g (95%) of diazoketone (**2**), which was used in the next step without further purification.

Diazoketone (**2**) (3 g, 6.3 mmol, 1 eq) was dissolved in the mixture of THF and water (55 mL; 10:1) in a 250 mL round bottom flask. Triethylamine (1.78 mL, 17.5 mmol, 2.8 eq) and silver trifluoroacetate (0.15 g, 0.7 mmol, 0.11 eq) were added, and stirring was continued for 30 min. The solution was diluted with diethyl ether (200 mL), followed by 5% NaHCO_3_ (200 mL). The white precipitate was filtrated off and combined with the aqueous phase. Its pH was adjusted to 2 with 2 M HCl, and the product was extracted with ethyl acetate (3 × 150 mL). The organic solution was washed with brine (100 mL) and dried over MgSO_4_. Evaporation of the solvent gave a white product, which, after purification by flash chromatography (hexane:ethyl acetate = 1:1; Rf = 0.3), yielded 1.5 g (51%) of **3**.

A total of 0.9 g of **3** was dissolved in dioxane (5 mL); 4N HCl/dioxane (10 mL) was added, and the mixture was stirred until the reaction was completed (LC-MS). The solid residue obtained after evaporation was suspended in propylene oxide (10 mL) and refluxed for 2 h until all chloride ions reacted with the silver nitrate solution. Then, diethyl ether was added, and the white precipitate was filtered and dried. The obtained zwitterionic product **4** (0.71 g, yield ~100%) was used in the next step without further purification.

In total, 0.71 g (1.92 mmol, 1 eq) of **4** was suspended in DCM (20 mL). N,O-bis(trimethylsilyl) acetamide (0.535 g, 2.5 mmol, 1.3 eq) was added and stirred for 30 min. Next, 4-methyltrityl chloride (0.562 g, 1.92 mmol, 1 eq) was added along with DIPEA (2.5 mmol, 0.44 mL, 1.3 eq). The reaction was kept overnight at r.t. and controlled with LC-MS. When completed, the solvent was evaporated, and the residue was dissolved in ethyl acetate (200 mL) and washed with 5% NaHCO_3_ (2 × 100 mL) and brine (100 mL). The organic layer was dried over MgSO_4_ and evaporated. The product was purified by flash chromatography (Rf = 0.2 in DCM:MeOH = 10:1), giving 0.67 g of the final product **5** with a 50% yield. ^1^H NMR spectrum (Figure S1) confirmed the structure.

### 4.3. Peptide Synthesis

Synthesis of linear precursors of cyclopeptides was performed by the standard manual solid-phase procedure on MBHA Rink-Amide resin (100–200 mesh, 0.8 mmol/g), using 9-fluorenylmethoxycarbonyl (Fmoc) protection for the α-amino groups of amino acids. N^ε^-amino group of (R)- and (S)-β^3^-Lys was protected by the 4-methyltrityl (Mtt), β-carboxy group of Asp by 2-phenyl-isopropyl ester (O-2 PhiPr) and hydroxy group of Tyr by t-butyl (t-Bu). Piperidine in DMF (20%) was used for the deprotection of Fmoc groups, and 2-(1H-benzotriazol-1-yl)-1,1,3,3-tetramethyluronium tetrafluoroborate (TBTU) was employed as a coupling agent and diisopropylethylamine (DIEA) as a neutralizing base. Fully assembled Fmoc-protected peptides were treated with 1% trifluoroacetic acid (TFA) in dichloromethane (DCM) to remove the side chain Mtt and O-2PhiPr protecting groups, followed by on-resin cyclization (TBTU). Cleavage from the resin was accomplished by treatment with TFA/triisopropylsilane (TIS)/water (95:2.5:2.5) for 3 h at room temperature.

Crude peptides were purified by preparative reversed-phase HPLC on a Vydac C_18_ column (10 μm, 22 × 250 mm), flow rate 2 mL/min, 20 min linear gradient from water/0.1% (*v*/*v*) TFA to 80% acetonitrile/20% water/0.1% (*v*/*v*) TFA. The purity of the final peptides was verified by analytical HPLC employing a Vydac C_18_ column (5 μm, 4.6 × 250 mm), flow rate 1 mL/min, and the same solvent system over 50 min. The purity of the obtained peptides was >95%. Calculated values for protonated molecular ions were in agreement with those determined by high-resolution mass spectroscopy with electrospray ionization (ESI-MS) (Table S1).

### 4.4. Opioid Receptor Binding Assays

The opioid receptor binding assays were performed according to the described method [39], using commercial membranes of Chinese Hamster Ovary (CHO) cells transfected with human opioid receptors. The binding affinities for MOR, DOR, and KOR were determined by radioligand competition analysis using [^3^H]DAMGO, [^3^H]deltorphin-2, and [^3^H]U-69593, respectively, as specific radioligands, respectively. Membrane preparations were incubated at 25 °C for 120 min with appropriate concentrations of a tested peptide in the presence of 0.5 nM radioligand in a total volume of 0.5 ml of 50 mM Tris/HCl (pH 7.4) containing bovine serum albumin (BSA) (1 mg/mL), bacitracin (50 µg/mL), bestatin (30 µM), and captopril (10 µM). Non-specific binding was determined in the presence of 1 µM naloxone. Incubations were terminated by the rapid filtration through the GF/B Whatman (Brentford, UK) glass fiber strips (pre-soaked for 2 h in 0.5% (*v*/*v*) polyethylamine) using Millipore Sampling Manifold (Billerica, MA, USA). The filters were washed three times with 4 ml of ice-cold Tris buffer solution. The bound radioactivity was measured in a Packard Tri-Carb 2100 TR liquid scintillation counter (Ramsey, MN, USA) after overnight extraction of the filters in 4 mL of a Perkin Elmer Ultima Gold scintillation fluid (Wellesley, MA, USA). Three independent experiments for each assay were carried out in duplicate. The data were analyzed by a nonlinear least square regression analysis computer program Graph Pad PRISM 6.0 (Graph Pad Software Inc., San Diego, CA, USA). The IC_50_ values were determined from the logarithmic concentration–displacement curves, and the values of the inhibitory constants (K_i_) were calculated according to the equation of Cheng and Prusoff [40].

### 4.5. Calcium Mobilization Assay

Calcium mobilization assay was performed, as reported in detail elsewhere [41], using CHO cells stably co-expressing human recombinant MOR or KOR and the C-terminally modified Gα_qi5_ and CHO cells co-expressing human recombinant DOR and the Gα_qG66Di5_ chimeric protein (a generous gift from Prof. Girolamo Calo, University of Padova, Italy). Cells were cultured in a culture medium consisting of Dulbecco’s MEM/HAMS F12 (1:1) supplemented with 10% fetal bovine serum, penicillin (100 IU/mL), streptomycin (100 µg/mL), L-glutammine (2 mM), fungizone (1 µg/mL), geneticin (G418; 200 µg/mL) and hygromycin B (100 µg/mL). Cell cultures were kept at 37 °C in 5% CO_2_/humidified air. Cells were seeded at a density of 50,000 cells/well into 96-well black, clear-bottom plates. After 24 h incubation, the cells were loaded with a medium supplemented with probenecid (2.5 mM), calcium-sensitive fluorescent dye Fluo-4 AM (3 µM), pluronic acid (0.01%), and HEPES (20 mM) and kept for 30 min at 37 °C. Then, the loading solution was aspirated, and 100 µL/well of assay buffer (HBSS supplemented with 20 mM HEPES, 2.5 mM probenecid, and 500 µM Brilliant Black) was added. After placing both plates (cell culture and compound plate) into the FlexStation II (Molecular Device, Union City, CA, USA), the on-line additions were carried out in a volume of 50 µL/well and the fluorescence changes were measured. Ligand efficacies, expressed as the intrinsic activity (α), were calculated as the E*_max_* ratio of the tested compound and the standard agonist. At least three independent experiments for each assay were carried out in duplicate.

Curve fittings were performed using Graph Pad PRISM 5.0 (GraphPad Software Inc., San Diego, CA, USA). Data have been statistically analyzed with one-way ANOVA followed by the Dunnett’s test for multiple comparisons; *p* values <0.05 were considered significant.

### 4.6. Quantum Chemical Calculations

One hundred conformers for compounds **RP-171** and **RP-172** were generated by an in-house Python script using the improved ETKDG method [42]. The compounds were protonated at the N-terminal nitrogen atom. The geometries were optimized in Gaussian09 [21] at the B3LYP/6-31G level in a gas phase or in water using the PCM solvent model. The resulting geometries were then reoptimized at the B3LYP/6-31G(d,p) level. Further attempts to increase the theory level were unsuccessful for the lack of convergence. Top conformers were subject to harmonic frequency calculations at the B3LYP/6-31G(d,p) level in order to ascertain that the geometries are minima (no imaginary frequencies) and to calculate thermochemical values.

### 4.7. Molecular Docking

One hundred conformers of **RP-171** and **RP-172** (obtained as described in Section 4.6) were docked into the activated structure of the MOR (PDB accession code: 6DDF [24], a complex of mu opioid receptor with Gi protein, with DAMGO peptide in the orthosteric binding site) using AutoDock 4.2.6 [25]. The ligands and the protein were processed in AutoDock Tools 4 [25]. The ligands’ side chains were allowed to rotate, and the receptor structure was kept rigid. The docking box was set around the position of the DAMGO molecule in the 6DDF structure [24]. The grids (82 × 78 × 104 points, with 0.375 Å spacing) were calculated with AutoGrid, and the docking was performed using Lamarckian Genetic Algorithm local searches according to the pseudo-Solis and Wets algorithm. Each docking consisted of 100 runs. The results were clustered, and the top scored solutions were visually inspected to examine their conformity to the known literature data on ligand MOR interactions [43]. Molecular graphics were prepared in Biovia Discovery Studio Visualizer [44].

### 4.8. Molecular Dynamics

The complexes of MOR with **RP-171** and **RP-172** (obtained by molecular docking, described in Section 4.7) were subject to molecular dynamics simulations in GROMACS 5.1.2 [45]. The complexes were embedded in a lipid bilayer of POPC molecules (128 molecules) solvated with water molecules (TIP3P type, 13,000 molecules) and supplied with ions (Na^+^ and Cl^−^, 0.154 M). These steps were performed with the CHARMM-GUI service [46]. CHARMM 36 force field was used for modeling the proteins, lipids, water, and ions. The ligands were modeled using CHARMM CGenFF [47].

The complexes were minimized and equilibrated, whereafter 100 ns production was performed (NPT ensemble, temperature = 303.15 K, integration step = 2 fs, cut-off scheme Verlet, Nose-Hoover thermostat, Parrinello–Rahman barostat, LINCS H-bonds constraints).

## Figures and Tables

**Table 1:** Receptor binding affinities (K_i_) of novel cyclic analogs at MOR, DOR, KOR.

No.	Sequence	K_i_ ^a^ [nM]		
		MOR	DOR	KOR
**RP-170**	Tyr-c[D-Lys-Phe-Phe-Asp]NH_2_	0.55 ± 0.02	198 ± 4.5	1.52 ± 0.20
**RP-171**	Tyr-c[(*R*)-β^3^-Lys-Phe-Phe-Asp]NH_2_	29 ± 4.32	>1000	420 ± 23
**RP-172**	Tyr-c[(*S*)-β^3^-Lys-Phe-Phe-Asp]NH_2_	950 ± 45	>1000	>1000

^a^ Binding affinities were determined by competitive displacement of the selective radioligands, [^3^H]DAMGO (MOR), [^3^H]deltorphin-2 (DOR), and [^3^H]U-69593 (KOR) using commercial membranes of CHO cells transfected with human opioid receptors. Values are expressed as mean ± SEM, n = 3.

**Table 2:** Effect of new analogs at human recombinant opioid receptors coupled with calcium signaling via chimeric G proteins.

Peptide	MOR		DOR		KOR	
	pEC_50_ (CL_95%_)	α ± SEM	pEC_50_ (CL_95%_)	α ± SEM	pEC_50_ (CL_95%_)	α ± SEM
**Dermorphin**	8.57 ± 0.07	1.00	inactive		inactive	
**DPDPE**	inactive		7.23 ± 0.22	1.00	inactive	
**Dynorphin A**	6.67 ± 0.50	0.83 ± 0.10	7.73 ± 0.27		9.04 ± 0.05	1.00
**RP-170**	8.93 ± 0.05	1.00	inactive		8.60 ± 0.14	1.00 ± 0.03
**RP-171**	6.87 ± 0.14	0.82 ± 0.02	inactive		5.99 ± 0.05	0.82 ± 0.05
**RP-172**	5.45 ± 0.91	0.45 ± 0.02	inactive		inactive	

Dermorphin, DPDPE, and dynorphin A were used as reference agonists for calculating intrinsic activity at MOR, DOR, and KOR, respectively: pEC50, aAgonist potency values; α, befficacy values; n ≥ 3.

## Data Availability

Not applicable.

## Supplementary Materials

The following are available online. Figure S1: H^1^ NMR spectrum of Fmoc-(*R*)- β^3^-Lys(Mtt); Figures S2 and S3: high-resolution mass spectra of analogs **RP-171** and **RP-172**; Figures S4–S6: LC-MS and MS^n^ analysis of analogs **RP-171** and **RP-172**; Figure S7: concentration–response curves of analogs in the functional assay; Figure S8: root mean square deviations of protein and ligand in the MD simulations; Table S1: physicochemical characterization of analogs 2–9; Table S2: total energies of top 15 conformers for **RP-171** and **RP-172**.
