# Supplementary material for: Synthesis, Pharmacological Evaluation, and Computational Studies of Cyclic Opioid Peptidomimetics Containing β3-Lysine
Source: Molecules. 2021 Dec 28;27(1):151. doi: 10.3390/molecules27010151 (PMC8747000; doi:10.3390/molecules27010151)
Supplement: Supplementary file 1 [file molecules-27-00151-s001.zip › molecules-1485541-supplementary.pdf]

Supplementary Materials

Synthesis, Pharmacological Evaluation, and Computational Studies of Cyclic Opioid Peptidomimetics Containing β³-Lysine

Karol Wtorek [1], Piotr F. J. Lipiński [2], Anna Adamska-Bartłomiejczyk [1], Justyna Piekielna-Ciesielska [1], Jarosław Sukiennik [3], Alicja Kluczyk [4] and Anna Janecka [1,*]

[1] Department of Biomolecular Chemistry, Medical University of Lodz, Mazowiecka 6/8, 92-215 Lodz, Poland; karol.wtorek@umed.lodz.pl (K.W.); anna.adamska@umed.lodz.pl (A.A.-B.); justyna.piekielna@umed.lodz.pl (J.P.-C.)
[2] Department of Neuropeptides, Mossakowski Medical Research Institute, Polish Academy of Sciences, Pawińskiego 5, 02-106 Warsaw, Poland; plipinski@imdik.pan.pl
[3] TriMen Chemicals Ltd., 92-318 Lodz, Poland; sukiennik@trimen.pl
[4] Faculty of Chemistry, University of Wroclaw, 50-383 Wroclaw, Poland; alicja.kluczyk@chem.uni.wroc.pl
* Correspondence: anna.janecka@umed.lodz.pl

Contents

- H¹ NMR spectrum of Fmoc-(*R*)- β³-Lys(Mtt) (Figure S1)……………………… ………p. S2
- Physicochemical characterization of analogs **2-9** (Table S1)…………………….… …p. S3
- High resolution mass spectra of analogs **RP-171** and **RP-172** (Figure S2 and S3).......... p. S4
- LC-MS and MSⁿ analysis of analogs **RP-171** and **RP-172** (Figure S4-S6)……………...p. S6
- Total energies of top 15 conformers for **RP-171** and **RP-172**, obtained at the
- B3LYP/6-31G(d,p) level (Table S2)…………………………………………………… p. S9
- Concentration-response curves of analogs in the functional assay (Figure S7) ....p. S10
- Root mean square deviations (RMSD) of protein (top) and ligand (bottom) positions for MD simulations of **RP-171** and **RP-172** with MOR Figure S8)……………………..p. S11
- References………………………………………………………………………….p. S12

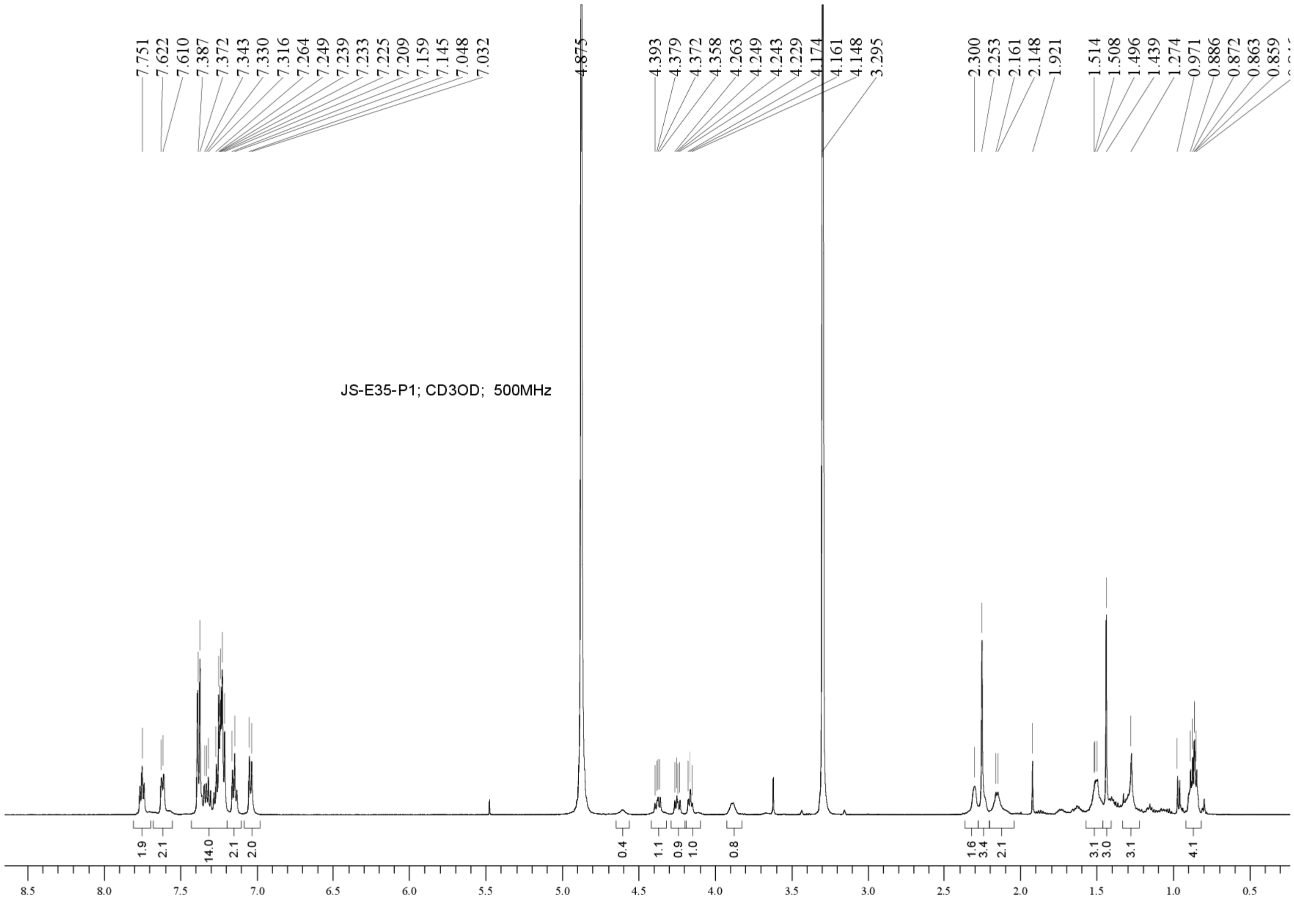

Figure S1. H[1] NMR spectrum of Fmoc-(*R*)- β[3]-Lys(Mtt).

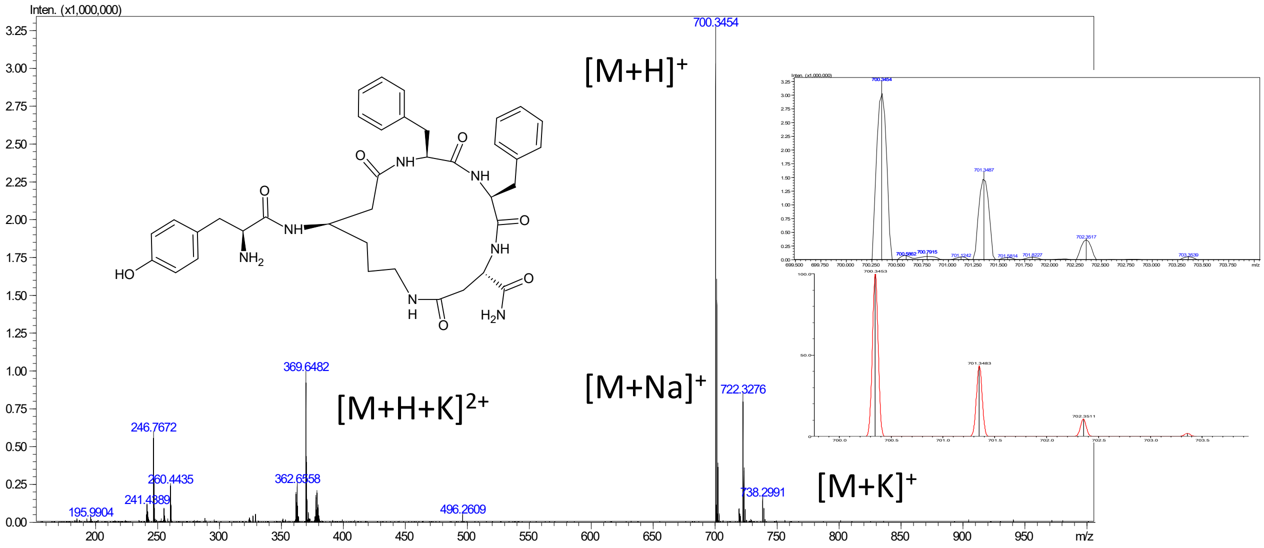

Figure S2. Analysis of peptide Tyr-c[(*R*)-β[3]Lys-Phe-Phe-Asp]NH_2 (**RP-171**) by mass spectrometry. High resolution MS spectrum. In inset, fragment of the experimental spectrum is compared with the simulated isotopic profile calculated for the expected molecular formula of protonated species $[M+H]^+$.

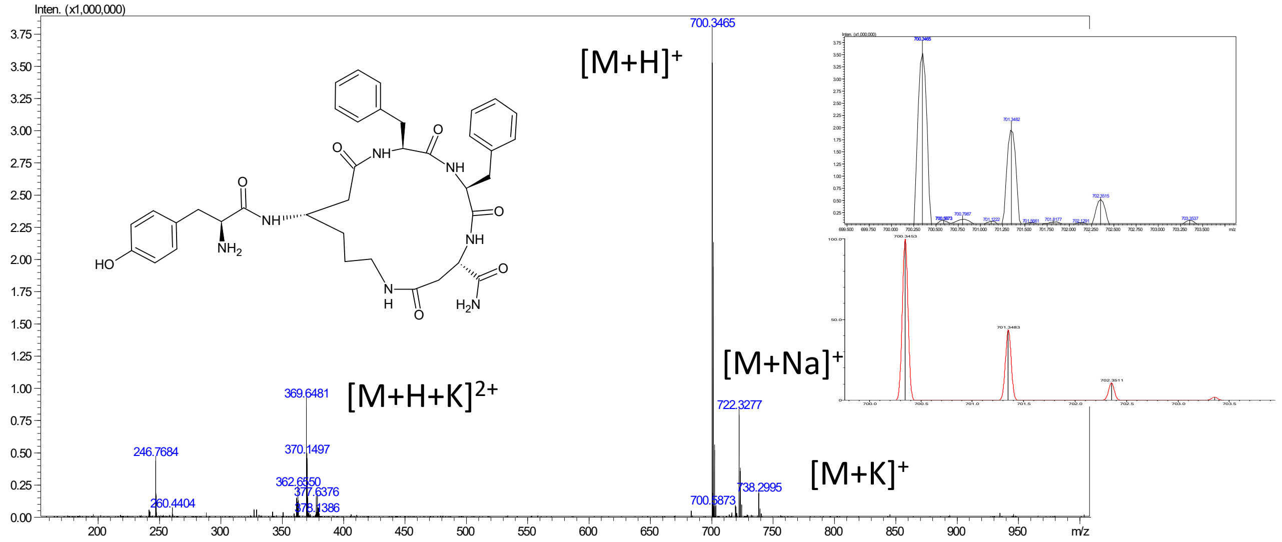

Figure S3. Analysis of peptide of peptide Tyr-c[(*S*)-β[3]Lys-Phe-Phe-Asp]NH_2 (**RP-172**).

High resolution MS analysis. In inset, fragment of the experimental spectrum is compared with the simulated isotopic profile calculated for the expected molecular formula of protonated species $[M+H]^+$.

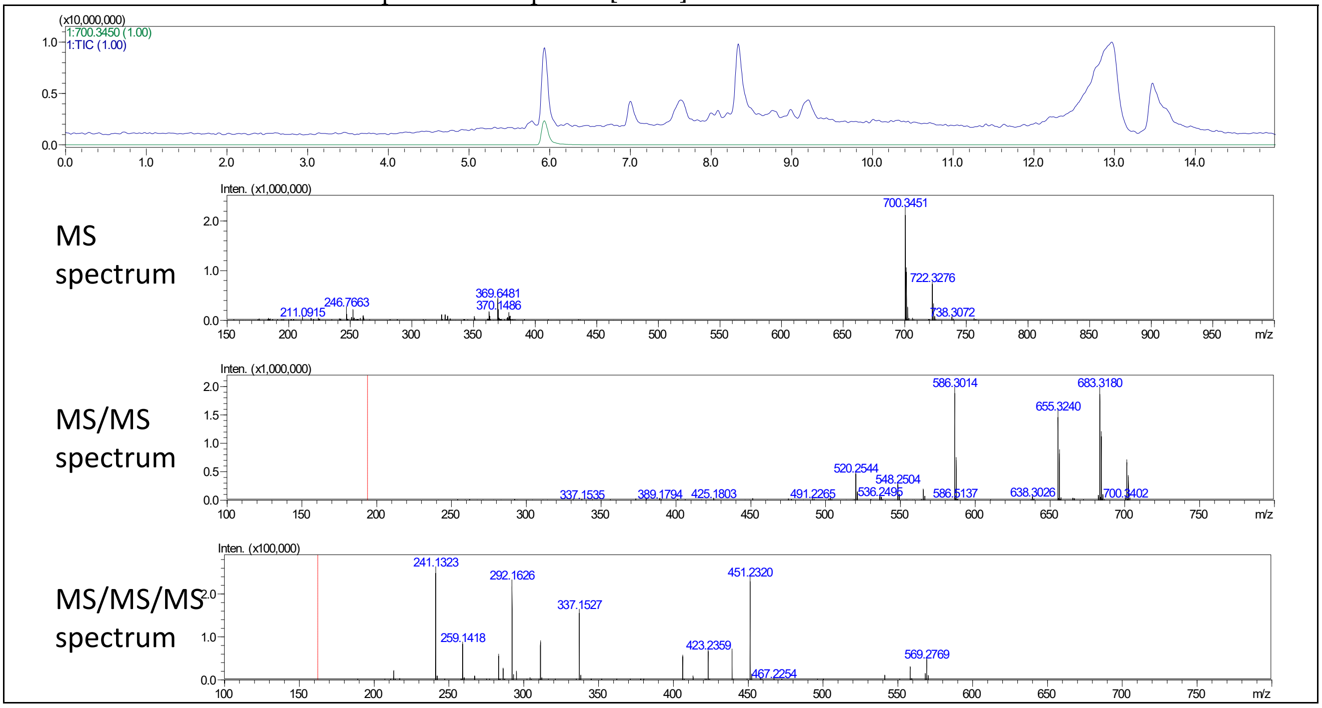

Figure S4. LC-MS analysis of peptide Tyr-c[(*R*)-β[3]Lys-Phe-Phe-Asp]NH_2 (**RP-171**). Top panel: total ion chromatogram (TIC, blue line) and extracted ion chromatogram for *m*/*z* 700.345 (XIC, green line). MS^n spectra recorded for the retention time in XIC. Kinetex Biphenyl column was used for chromatographic separation.

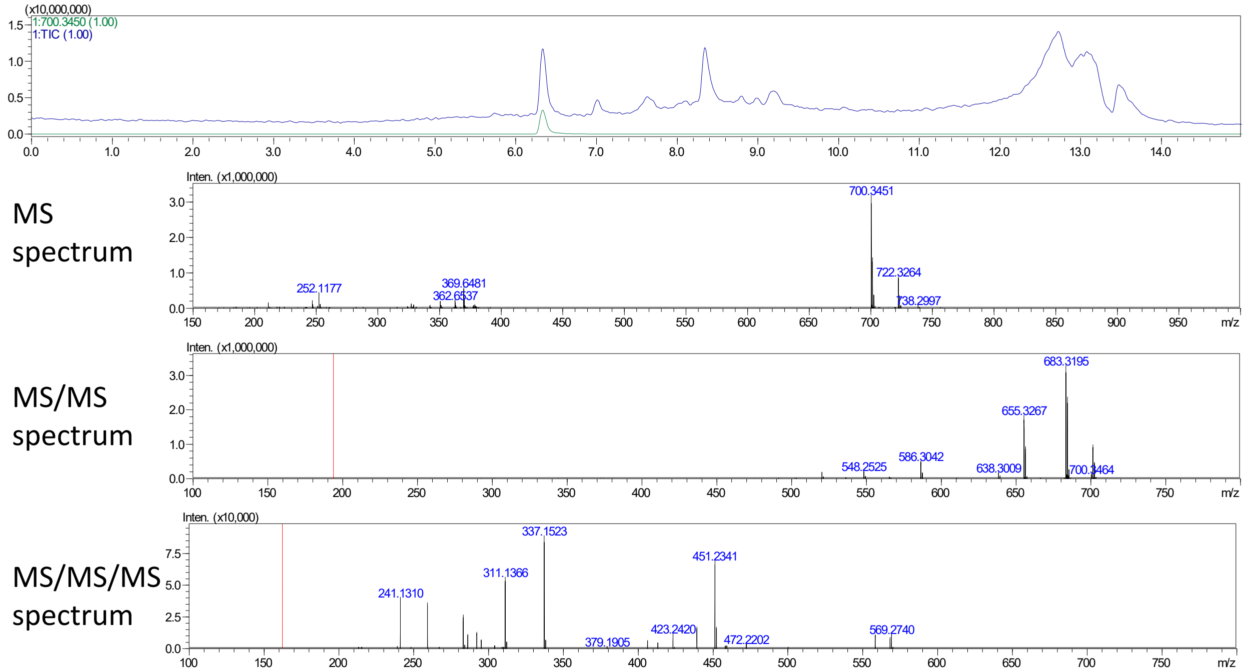

Figure S5. LC-MS analysis of peptide Tyr-c[(*S*)-β[3]Lys-Phe-Phe-Asp]NH2 (**RP-172**). Top panel: total ion chromatogram (TIC, blue line) and extracted ion chromatogram for *m*/*z* 700.345 (XIC, green line). MS^n spectra recorded for the retention time in XIC. Kinetex Biphenyl column was used for chromatographic separation.

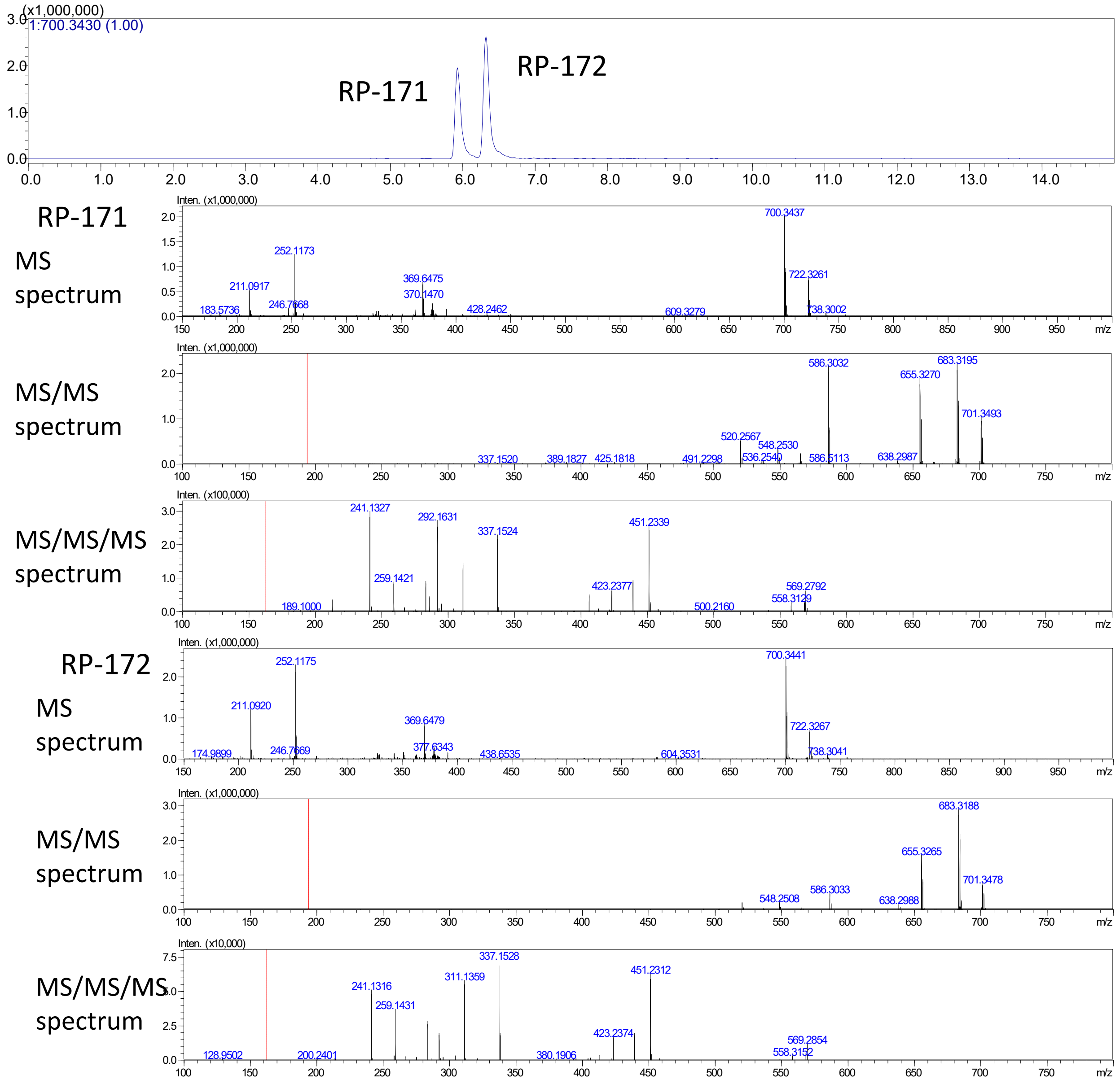

Figure S6. LC-MS analysis of peptide mixture, containing Tyr-c[(*R*)-β³-Lys-Phe-Phe-Asp]NH₂ (**RP-171**) and Tyr-c[(*S*)-β³Lys-Phe-Phe-Asp]NH₂ (**RP-172**). Top panel: total ion chromatogram (TIC, blue line) and extracted ion chromatogram for *m*/*z* 700.345 (XIC, green line). MS^n spectra recorded for the retention times in XIC. Kinetex Biphenyl column was used for chromatographic separation.

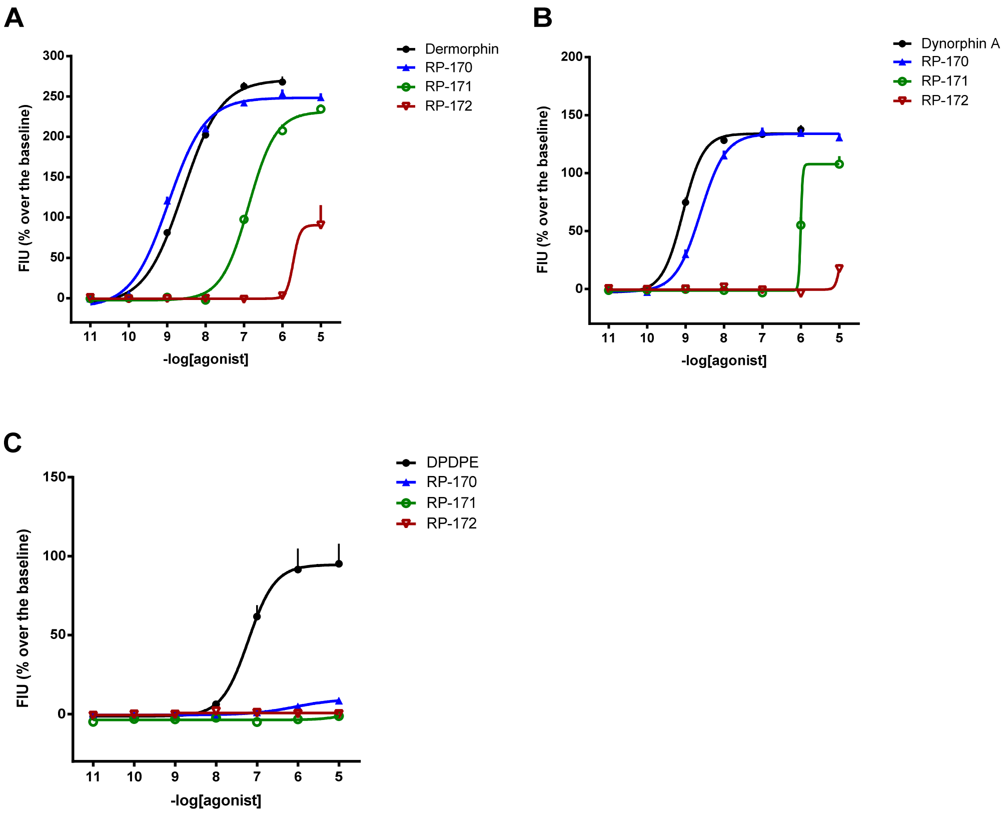

Figure S7. Concentration-response curves to reference agonists and tested peptides in calcium mobilization experiments performed in CHO cells stably co-expressing the human MOR (Panel A) or KOR (Panel B) and the C-terminally modified $G\alpha_{qi5}$ and CHO cells co-expressing DOR (Panel C) and the $G\alpha_{qG66Di5}$ protein. Data are the mean±SEM of at least 4 separate experiments performed in duplicate.

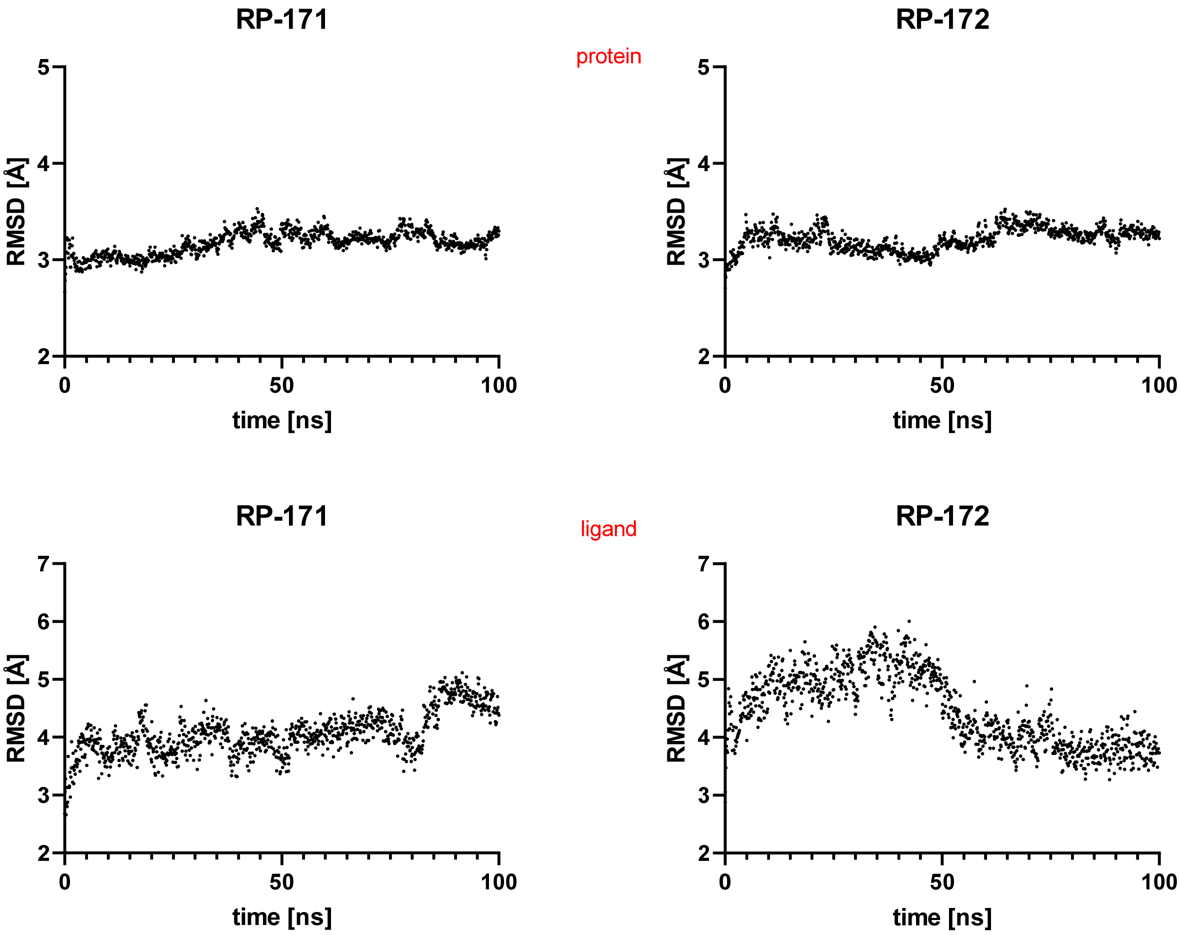

Figure S8. Root mean square deviations (RMSD) of protein (top) and ligand (bottom) positions for MD simulations of **RP-171** and **RP-172** with MOR.

Table S1. Physicochemical characterization of analogs.

No.	Sequence	Formula	m/z $[M + H]^{+a}$		HPLC t_R[b]
			Calcd	Obsd	[min]
**RP-171**	Tyr-c[(*R*)-β[3]Lys-Phe-Phe-Asp]NH_2	$C_{37}H_{44}N_7O_6$	700.3453	700.3454	14.05
**RP-172**	Tyr-c[(*S*)-β[3]Lys-Phe-Phe-Asp]NH_2	$C_{37}H_{44}N_7O_6$	700.3453	700.3465	14.36

[a] Observed by ESI MS^+ ionization. Mass spectra of peptides were recorded using Shimadzu IT-TOF mass spectrometer equipped with ESI ion source and operated in positive ion mode.

[b] RP-HPLC was performed on a Vydac C_{18} column (5 μm, 4.6 x 250 mm) using the solvent system of 0.1% TFA in water (A) and 80% acetonitrile in water containing 0.1% TFA (B) and a linear gradient of 0–100% solvent B over 50 min, with a flow rate of 1 mL/min.

In the MS^3 experiment, the 292 *m*/*z* ion corresponds to Tyr-β[3]-Lys fragment, 451 *m*/*z* and 337 *m*/*z* ion are probably formed by removal of Tyr from the 586 *m*/*z* ion and internal cyclization, whereas 311 *m*/*z* ion represents fragment containing Phe and Tyr residues, formed during internal rearrangement, a frequent occurrence in MS analysis of cyclic peptides [1,2,3,4]. There are at least two pathways in 586 *m*/*z* ion fragmentation, with the internal cyclization as well as rearrangement leading to Tyr-Phe fragment formation predominant in **RP-172**, probably resulting from a more stable structure due to orientation of Tyr residue, although a final assignment requires additional MS^n studies.

Table S2. Total energies of top 15 conformers for **RP-171** and **RP-172**, obtained at the B3LYP/6-31G(d,p) level.

	Conformer code	Total energy (Hartrees)
	**RP-171**	
**1**	RP-171__31	-2347.500135
**2**	RP-171__43	-2347.500042
**3**	RP-171__25	-2347.494393
**4**	RP-171__39	-2347.493544
**5**	RP-171__12	-2347.489088
**6**	RP-171__92	-2347.487881
**7**	RP-171__56	-2347.485198
**8**	RP-171__60	-2347.484992
**9**	RP-171__96	-2347.483326
**10**	RP-171__30	-2347.479364
**11**	RP-171__27	-2347.474592
**12**	RP-171__3	-2347.474055
**13**	RP-171__35	-2347.473866
**14**	RP-171__0	-2347.473614
**15**	RP-171__72	-2347.472941
	**RP-172**	
**1**	RP-172__20	-2347.502704
**2**	RP-172__41	-2347.498904
**3**	RP-172__84	-2347.498412
**4**	RP-172__62	-2347.498134
**5**	RP-172__11	-2347.496409
**6**	RP-172__8	-2347.495252
**7**	RP-172__31	-2347.493671
**8**	RP-172__37	-2347.493462
**9**	RP-172__56	-2347.493301
**10**	RP-172__49	-2347.493077
**11**	RP-172__6	-2347.492747
**12**	RP-172__51	-2347.492158
**13**	RP-172__71	-2347.491597
**14**	RP-172__70	-2347.49103
**15**	RP-172__64	-2347.490503

References

1. Ngoka, L. C. M.; Gross, M. L. Multistep Tandem Mass Spectrometry for Sequencing Cyclic Peptides in an Ion-Trap Mass Spectrometer. *J. Am. Soc. Mass. Spectrom.* **1999**, *10*, 732-746.
2. Jia, C.; Qi, W.; He, Z.; Qiao, B. Sequencing peptides by electrospray ion-trap mass spectrometry: A useful tool in synthesis of Axinastatin 3. *Cent. Eur. J. Chem.* **2006**, *4*, 620-631.
3. Stefanowicz, P. Electrospray mass spectrometry and tandem mass spectrometry of the natural mixture of cyclic peptides from linseed, *Eur. J. Mass. Spectrom.* **2004**, *10*, 665-671.
4. Eckart, K. Mass spectrometry of cyclic peptides. *Mass Spectrom. Rev.* **1994**, *13*, 23-55.
